# Development and validation of predictive models for the hemodialysis adequacy index (Kt/V) using multiple linear regression and machine learning algorithms

**DOI:** 10.3389/fdgth.2026.1771806

**Published:** 2026-10-12

**Authors:** Le-le Li, Rui-feng Xu, Liang-liang Huang, Bing-ru Lu, Shu-ting Zhang, Ya Tuo, Hui-qing Liu, Rui Ren, Shu-fang Wang, Ming-xia Sun, Xiao-tong Zhang, Jian-rong Zhao, Lei Yu, Yan Meng

**Affiliations:** 1 Department of Nephrology, The Affiliated Hospital of Inner Mongolia Medical University, Hohhot, China; 2 Department of Nephrology, Hohhot First Hospital, Hohhot, China; 3 Department of Information Engineering, Ordos Vocational College, Ordos, China; 4 Hemodialysis Room, Togtoh County Hospital, Hohhot, China; 5 Department of Nephrology, Inner Mongolia Autonomous Region Hospital of Traditional Chinese Medicine, Hohhot, China; 6 The First Department of Specialty Medicine, Inner Mongolia Corps Hospital of the Chinese People’s Armed Police Force, Hohhot, China; 7 Department of Internal Medicine, The First Hospital of Inner Mongolia Autonomous Region Prison Administration, Hohhot, China; 8 Department of Nephrology, Inner Mongolia People’s Hospital, Hohhot, China

**Keywords:** clinical prediction model, hemodialysis adequacy, machine learning algorithms, multiple linear regression (MLR), urea clearance index (Kt/V)

## Abstract

**Background and objective:**

Kt/V is a widely used indicator for assessing hemodialysis (HD) adequacy, but conventional measurement methods require blood sampling and are impractical for large-scale or real-time use. This study aimed to develop and validate predictive models for Kt/V using multiple linear regression (MLR) and machine learning algorithms, enabling clinicians to estimate dialysis adequacy before a session begins using simple, non-invasive parameters.

**Methods:**

A total of 1,017 eligible patients were enrolled from seven HD centers in Hohhot City, China, and divided into training (*n* = 677), internal (*n* = 220), and external validation (*n* = 120) sets. From 26 candidate variables, eight clinically meaningful derived features were constructed, and a multi-algorithm fusion strategy integrating *F* test, Mutual Information, Random Forest importance, XGBoost importance, LASSO coefficients, Permutation Importance, Recursive Feature Elimination, and Boruta was employed for feature selection. Five final features (body surface area, pre-dialysis weight, standardized blood flow, sex, and circulating blood volume) were selected. Eight point-prediction models (MLR, XGBoost, LightGBM, Random Forest, Gradient Boosting, KNN, SVM, and BPNN), a simple average ensemble of the three top-performing models, and two probabilistic prediction methods (LightGBM Quantile Regression and NGBoost) were developed. Hyperparameter tuning was performed using GridSearchCV with 5-fold cross-validation. Model performance was evaluated using R^2^, RMSE, and MAE on internal and external validation sets.

**Results:**

On external validation, the SVM model achieved the highest R^2^ (0.5931) and lowest RMSE (0.1633) and MAE (0.1214) among individual models, followed by Gradient Boosting (R^2^ = 0.5748, RMSE = 0.1670, MAE = 0.1276) and XGBoost (R^2^ = 0.5681, RMSE = 0.1683, MAE = 0.1288). The ensemble model achieved the best overall performance (R^2^ = 0.5911, RMSE = 0.1637, MAE = 0.1240). Calibration assessment revealed all models were well-calibrated without requiring post-hoc adjustment. For probabilistic prediction, NGBoost provided well-calibrated 80% prediction intervals (PICP = 82.27%) with narrower intervals than LightGBM Quantile Regression. SHAP analysis identified BSA, standardized blood flow, and gender as the most influential predictors.

**Conclusion:**

Machine learning models, particularly SVM, the ensemble model, and NGBoost, can provide accurate, interpretable, and uncertainty-aware predictions of Kt/V using non-invasive, readily available parameters. The development of a local standalone executable application further facilitates clinical translation, enabling pre-dialysis prediction and real-time prescription adjustment. Prospective validation in diverse populations is warranted to confirm generalizability.

## 1 Introduction

Chronic kidney disease (CKD) has an increasing prevalence in China and throughout the world, and a recent study estimated the global prevalence rate was 14.3% (1). Thus, it is necessary to provide better treatments for CKD to increase the survival time and improve the quality of life in these patients (2). CKD is most common in patients with diabetes or high blood pressure, and it can progress at different rates to end-stage renal disease (ESRD) (3). Renal replacement therapy (RRT), including hemodialysis (HD), peritoneal dialysis, and kidney transplantation, is the only effective treatment for ESRD. Due to its convenience, safety, and effectiveness, HD has become the main method of RRT for patients with ESRD (4).

Adequate HD can be broadly defined (5) as achieving and maintaining a good clinical status through dialysis treatment (based on improved blood pressure, intravascular volume, cardiac function, hemoglobin level, appetite, and quality-of-life), and more narrowly defined as a satisfactory clearance rate of small-molecule solutes. Kt/V (known as the “urea clearance index” or the “hemodialysis adequacy index”), is the most commonly used quantitative indicator of the clearance rate of small-molecule solutes (6). Inadequate HD is associated with clinical complications, poor quality of life, and poor survival rates (7, 8). Therefore, it is important for clinicians to assess dialysis adequacy in patients receiving maintenance hemodialysis (MHD), and Kt/V is widely used as an indicator to formulate and adjust dialysis treatment plans.

Currently, there are two internationally recognized methods for determining Kt/V. The first method is based on the urea kinetic model proposed by Gotch and Sargent, which calculates Kt/V by measuring the pre- and post-dialysis plasma urea nitrogen levels from blood samples (9). However, this method requires collection of multiple blood samples, which can exacerbate anemia and patient discomfort, and can have an unacceptable economic burden. The results from a sample after a single dialysis session can be inadequate for formulation of a dialysis plan using this method. The second method, online clearance monitoring, uses parameters from the dialysis machine that are related to urea clearance (10). However, many HD machines cannot record these data, and no large-scale clinical studies have validated the accuracy of machine-based online detection. Therefore, the clinical practice guidelines for CKD (NKF-K/DOQI, 2006) do not recommend use of components built into dialysis machines for detection of dialysis adequacy. For these reasons, both of these methods can be problematic in clinical settings.

Clinical prediction models, defined as statistical or machine learning models that are based on the phenotypic characteristics of a disease, can predict the probability of the future occurrence of an outcome or a specific numerical value or indicator in a population with certain clinical features (11). Studies of other diseases utilized similar models to predict the risk of disease onset and progression, and treatment outcomes (11, 12). The purpose of the present study was to develop and validate predictive models for HD adequacy (Kt/V). We employed multiple linear regression (MLR), seven machine learning algorithms for point prediction (XGBoost, LightGBM, Random Forest, Gradient Boosting, KNN, SVM, and BPNN), an ensemble model combining three of these algorithms, and two probabilistic prediction algorithms (LightGBM Quantile Regression and NGBoost). The predictive performance of all models was then compared using internal and external validation sets. Our aim was to develop a reliable model for prediction of Kt/V that can be used by practicing clinicians, by helping them to formulate HD treatment plans in a more timely and scientifically informed manner, and thereby improve patient quality-of-life and long-term survival.

## 2 Data and methods

### 2.1 Data preparation

#### 2.1.1 Study design and study population

This was a multicenter, prospective observational study conducted across seven hemodialysis (HD) centers in Hohhot City, Inner Mongolia, China, from January 2022 to September 2022. The participating centers included the Affiliated Hospital of Inner Mongolia Medical University, Hohhot First Hospital, Inner Mongolia People's Hospital, Togtoh County Hospital, Inner Mongolia Armed Police General Hospital, Inner Mongolia Traditional Chinese Medicine Hospital, and the First Hospital of Inner Mongolia Autonomous Region Prison Administration.

The study was approved by the ethics committee of the Affiliated Hospital of Inner Mongolia Medical University (S.2021086) and was performed in accordance with the ethical standards in the 1964 Declaration of Helsinki and its later amendments. Written informed consent was obtained from all participants or their legal representatives prior to enrollment.

A total of 1,200 consecutive maintenance hemodialysis (MHD) patients were initially screened. Inclusion criteria were: (1) age between 18 and 80 years; (2) diagnosis of chronic kidney disease (CKD) requiring MHD; (3) clear consciousness and ability to communicate normally; (4) willingness to participate and provision of informed consent. Exclusion criteria were: (1) presence of language, visual, auditory, or intellectual impairment preventing participation; (2) age <18 or >80 years; (3) New York Heart Association Functional Class IV; (4) frequent recent angina attacks; (5) resting oxygen saturation <90%; (6) any other serious illnesses; or (7) refusal to participate. Patients with missing or incomplete data were excluded from the final analysis. After applying the inclusion and exclusion criteria, 1,017 eligible patients were enrolled.

#### 2.1.2 Data collection and outcome definition

The following demographic and clinical information was collected: gender, age, height, ethnicity, pre-dialysis weight, post-dialysis weight, HD vintage, dialysis duration per session, dialysis frequency, dialyzer membrane surface area and material, dialysate sodium concentration, blood flow rate, dialysate flow rate, access type, anticoagulant type, primary disease, and laboratory parameters including pre-dialysis and post-dialysis serum urea, hemoglobin (Hb), albumin (ALB), triglycerides (TG), total cholesterol (TC), C-reactive protein (CRP), platelets, parathyroid hormone (PTH), serum phosphorus, serum calcium, and serum potassium.

Serum urea concentration was measured before and after dialysis and the data were used to measure Kt/V. Before dialysis, blood was collected from the arterial end of the dialysis circuit without heparin or saline; after dialysis, ultrafiltration was stopped and the blood was collected from the arterial end of the dialysis circuit after the blood flow rate decreased to 100 mL/min for 15 s.

The primary outcome variable was Kt/V, calculated using the second generation Daugirdas formula (Equation 1):

Kt/V=−ln(R−0.008×t)+(4−3.5×R)×UF/W (1)

where R is the ratio of pre-dialysis to post-dialysis serum urea, t is the dialysis duration (hours), UF is the ultrafiltration volume (liters), and W is the post-dialysis body weight (kg).

### 2.2 Dataset partitioning and data preprocessing

#### 2.2.1 Dataset partitioning

Eligible patients were divided into three independent datasets following a stratified approach: (1) Training set (*n* = 677): used for feature selection, hyperparameter tuning, and final model training; (2) Internal validation set (*n* = 220): strictly withheld from all training and tuning procedures; used only once after model finalization for initial unbiased performance assessment; (3) External validation set (*n* = 120): sourced from three different institutions not represented in the training set; used as the ultimate independent test set to evaluate generalizability.

This strict three-way split ensured that the internal and external validation sets were never exposed to any model development step, providing an unbiased evaluation of model performance.

#### 2.2.2 Missing data imputation

Missing values in continuous predictor variables were imputed using the median value calculated from the training set only. Missing values in categorical variables were imputed using the mode from the training set. The same imputation values derived from the training set were applied to the internal and external validation sets to prevent data leakage.

#### 2.2.3 Feature standardization

To ensure compatibility with distance-based and gradient-based algorithms (SVM, KNN, BPNN), all continuous predictor variables were standardized using Z-score normalization.

Specifically, the mean (*μ*) and standard deviation (*σ*) for each continuous feature were calculated exclusively from the training set. These training-set parameters were then applied to transform the training, internal validation, and external validation sets (Equation 2):

x_standardized=(x−μ_train)/σ_train (2)

This approach simulates a real-world deployment scenario, where new patients are standardized using fixed parameters derived from the development data, and prevents any information leakage from the validation sets.

### 2.3 Feature engineering

#### 2.3.1 Several constructed derived features

To capture clinically meaningful information beyond the raw measurements, we constructed several derived features from the original variables:

1. Circulating blood volume (mL) = actual blood flow rate (mL/min) × actual dialysis duration (h) × 60;
2. Standardized blood flow (mL/min/m^2^) = actual blood flow rate (mL/min)/body surface area (m^2^);
3. Ultrafiltration rate (L/h) = actual ultrafiltration volume (L)/actual dialysis duration (h);
4. Pulse pressure (mmHg) = pre-dialysis systolic BP—pre-dialysis diastolic BP;
5. Mean arterial pressure (mmHg) = diastolic BP + (systolic BP—diastolic BP)/3;
6. Lipid accumulation index = total cholesterol (mmol/L) × triglycerides (mmol/L);
7. Nutritional index = albumin (g/L)/hemoglobin (g/L);
8. Weight to BSA ratio = pre-dialysis weight (kg)/body surface area (m^2^).

All original and constructed features were considered as candidate predictors for subsequent screening.

#### 2.3.2 Feature selection

We employed a multi-algorithm fusion strategy to identify the most robust set of predictors, integrating eight complementary feature selection methods that span different methodological paradigms:

1. *F* test (filter method, for linear associations);
2. Mutual Information (filter method, for non-linear associations);
3. Random Forest importance (embedded method, tree-based with interaction capture);
4. XGBoost importance (embedded method, gradient-boosting with gain-based importance);
5. LASSO coefficients (embedded method, L1-regularized linear model for sparse selection);
6. Permutation Importance (model-agnostic diagnostic, assessing drop in performance after shuffling);
7. RFE (Recursive Feature Elimination) (wrapper method, iteratively removing the least important features);
8. Boruta (wrapper method, random forest-based with shadow feature comparison) (13).

For each candidate variable, the ranks from the first seven algorithms were averaged to generate a consensus importance ranking. The Boruta algorithm categorizes each candidate variable into one of three classes, confirmed, rejected, or tentative, based on its importance relative to shadow features, allowing the investigator to decide whether a variable should be retained for subsequent model development. This ensemble-based approach ensures that a variable is retained only if it is consistently important across fundamentally different methodological paradigms, linear vs. non-linear, filter vs. embedded vs. wrapper, and tree-based vs. model-agnostic.

#### 2.3.3 Outlier removal

To prevent extreme values from unduly influencing model training, we performed outlier removal based on the Kt/V distribution in the training set. Specifically, samples with |Z| > 3 (i.e., beyond 3 standard deviations from the mean) in the training set were identified as extreme outliers and excluded prior to model training. This step was performed after feature selection to ensure that the model learned the dominant physiological patterns rather than fitting to extreme cases.

### 2.4 Model development for point prediction

#### 2.4.1 Candidate models

We developed and compared eight different predictive models using the five selected features:

1. MLR (Multiple Linear Regression), served as the baseline linear model;
2. XGBoost, an extreme gradient boosting algorithm based on decision trees;
3. LightGBM, a lightweight gradient boosting framework with leaf wise tree growth;
4. RandomForest, a classical bagging ensemble of decision trees;
5. GradientBoosting, a sequential boosting algorithm with decision trees as base learners;
6. KNN (K Nearest Neighbors), a distance-based non parametric regression method;
7. SVM (Support Vector Machine), with a radial basis function (RBF) kernel for non linear regression;
8. BPNN (Back Propagation Neural Network), a multi layer perceptron with two hidden layers.

All models were implemented in Python 3.10 using scikit-learn, XGBoost, and LightGBM libraries.

#### 2.4.2 Hyperparameter tuning

Hyperparameter optimization was performed using GridSearchCV with 5-fold cross-validation on the training set. The objective function was to maximize the average R^2^ across the 5 folds.

For each model, we pre-defined a search space of candidate hyperparameters. The full hyperparameters search spaces are provided in Supplementary Table S1.

Key examples of the tuned hyperparameters included:

1. XGBoost: n_estimators, max_depth, learning_rate, subsample, colsample_bytree, gamma, reg_lambda, min_child_weight
2. SVM: C, gamma, epsilon (RBF kernel)
3. BPNN: hidden_layer_sizes, activation, alpha, learning_rate_init
4. KNN: n_neighbors, weights, p

After the optimal hyperparameters were identified via GridSearchCV, the final model for each algorithm was retrained on the full training set using these optimal parameters.

For boosting-based models (XGBoost, LightGBM, GradientBoosting), early stopping was applied during training to prevent overfitting: training was terminated if the validation loss did not improve for 30 consecutive rounds.

#### 2.4.3 Ensemble model

In addition to the individual models, we constructed a simple average ensemble combining the predictions of the top performing models (SVM, GradientBoosting, and XGBoost), which were selected based on their superior test set RMSE and MAE.

The ensemble prediction was calculated as (Equation 3):

$$\hat{y}\_ensemble=(\hat{y}\_SVM+\hat{y}\_GradientBoosting+\hat{y}\_XGBoost)/3$$ (3)

This ensemble approach leverages the complementary strengths of geometrically based (SVM) and tree based (GradientBoosting, XGBoost) models to achieve more robust and stable predictions.

#### 2.4.4 Model calibration assessment

To assess whether the predictions from the three selected individual models (SVM, GradientBoosting, and XGBoost) and their simple average ensemble exhibited systematic bias (i.e., consistent over- or under-estimation), we performed a calibration diagnosis on the internal validation set.

A linear calibration model was fitted for each candidate model (Equation 4):

$$\hat{y}\_calibrated=a\times \hat{y}\_raw+b$$ (4)

where ŷ_raw is the original model prediction, a is the calibration slope, and b is the calibration intercept. A perfectly calibrated model would have a slope of 1 and an intercept of 0. The residual distribution (residual = true Kt/V−predicted Kt/V) was examined using: (1) residual-vs.-predicted scatter plots; (2) histograms of residuals; (3) true-vs.-predicted scatter plots; and (4) Q-Q plots for normality.

To quantify the potential benefit of *post-hoc* calibration, we calculated the improvement in MAE (mean absolute error) and the change in R^2^ after applying the linear calibration transformation. A model was considered well-calibrated and not requiring *post-hoc* adjustment if the MAE improvement was less than 0.005, as this threshold was deemed clinically negligible for Kt/V prediction.

#### 2.4.5 Model interpretability (SHAP analysis)

To explain the contribution of each feature to the model predictions, we performed SHAP (SHapley Additive exPlanations) analysis for the top performing models (SVM and GradientBoosting).

SHAP values quantify the marginal contribution of each feature to the prediction for each individual patient, providing both global feature importance rankings and local explanations for individual predictions.

### 2.5 Performance evaluation for point prediction models

Model performance was assessed using the following metrics: (1) R^2^ (Coefficient of Determination), the proportion of variance in Kt/V explained by the model; (2) RMSE (Root Mean Squared Error), the square root of the average squared prediction error, sensitive to large errors; (3) MAE (Mean Absolute Error), the average absolute prediction error, interpretable in the original Kt/V units; (4) MAPE (Mean Absolute Percentage Error), the average percentage error. All metrics were computed separately for the internal validation set (initial unbiased assessment) and the external validation set (final generalizability assessment).

To evaluate overfitting, we compared performance between the training set (from cross validation) and the validation sets. A small performance gap indicates that the model does not suffer from substantial overfitting.

### 2.6 Development and performance evaluation for probabilistic prediction models

To address the editor's request for prediction uncertainty estimation, we implemented two probabilistic prediction methods capable of generating prediction intervals alongside point estimates:

1. LightGBM Quantile Regression, direct modeling of conditional quantiles (0.05, 0.50, 0.95) using the quantile regression objective. This approach makes no distributional assumptions and can handle asymmetric error distributions.
2. NGBoost (Natural Gradient Boosting), a probabilistic gradient boosting framework that models the parameters of a log normal distribution for log transformed Kt/V. It outputs a full probability distribution from which prediction intervals and probability density curves can be derived.

The performance of probabilistic models was evaluated using: (1) PICP (Prediction Interval Coverage Probability), the proportion of true values falling within the predicted interval (ideal: close to the nominal confidence level); (2) MPIW (Mean Prediction Interval Width), the average width of the prediction intervals (narrower is better); (3) CWC (Coverage Width Criterion), a composite metric balancing PICP and MPIW (lower is better); (4) CRPS (Continuous Ranked Probability Score), a strictly proper scoring rule evaluating the entire predictive distribution (lower is better); (5) Pinball Loss, for quantile regression models; (6) NLL (Negative Log Likelihood), for NGBoost.

### 2.7 Bridging *post-hoc* development and pre-dialysis application

A critical point warrants explicit clarification regarding the intended clinical use of our model. During model development, we used post-dialysis parameters (actual dialysis duration, actual blood flow rate, and actual ultrafiltration volume) because these were the only objectively documented values available in our retrospective dataset.

In routine clinical practice, however, the dialysis prescription is determined by the clinician before the session begins, i.e., the planned dialysis duration, planned blood flow rate, and planned ultrafiltration volume are entered into the dialysis machine prior to treatment initiation. Importantly, due to standardized treatment protocols and stable machine performance, the prescribed parameters are virtually identical to the delivered actual parameters in the vast majority of treatments. In our dataset, the mean absolute difference between prescribed and actual values was negligible (<2% for all three parameters), confirming this consistency.

Therefore, although the model was developed using *post-hoc* actual values, its intended real-world application is pre-dialysis prediction, i.e., clinicians can input the planned prescription parameters (which are known before dialysis starts) into the identical model to obtain a reliable prediction of the Kt/V that would be achieved if that prescription is followed. This enables real-time, pre-emptive adjustment of the dialysis prescription, for example, increasing blood flow rate or extending dialysis duration, before the session actually begins, a functionality that the Daugirdas formula (which requires post-dialysis urea measurements by blood sampling and testing) cannot provide.

### 2.8 Development of a clinical application interface

To facilitate clinical adoption, we developed a local standalone executable application that implements the final ensemble model and the NGBoost probabilistic predictor. The application was packaged using PyInstaller and is distributed as a self-contained file package that can be executed offline without network connection. The interface accepts five input parameters (sex, height, pre-dialysis weight, blood flow rate, dialysis duration) and returns point predictions, prediction intervals, and probability density curves.

### 2.9 Statistical analysis and software

All statistical analyses and model development were performed using Python 3.10 (Python Software Foundation) with the following core libraries: pandas 2.3.3, numpy 2.2.6, scikit learn 1.7.2, xgboost 3.2.0, lightgbm 4.6.0, boruta 0.4.3, matplotlib 3.10.9, seaborn 0.13.2, shap 0.49.1, and properscoring 0.1.

Descriptive statistics are presented as mea*n* ± standard deviation for normally distributed continuous variables, median (Q1, Q3) for non-normally distributed variables, and frequencies (percentages) for categorical variables. The Shapiro–Wilk test was used to assess normality.

All code used for data preprocessing, feature selection, model training, hyperparameter tuning, and evaluation are available. The final trained models are provided from the corresponding author upon reasonable request.

## 3 Results

### 3.1 Study population and baseline characteristics

A total of 1,017 eligible patients were enrolled from seven hemodialysis centers. The training set comprised 677 patients (66.6%), the internal validation set 220 patients (21.6%), and the external validation set 120 patients (11.8%). Figure 1 shows the flow chart for disposition of patients.

**Figure 1:**
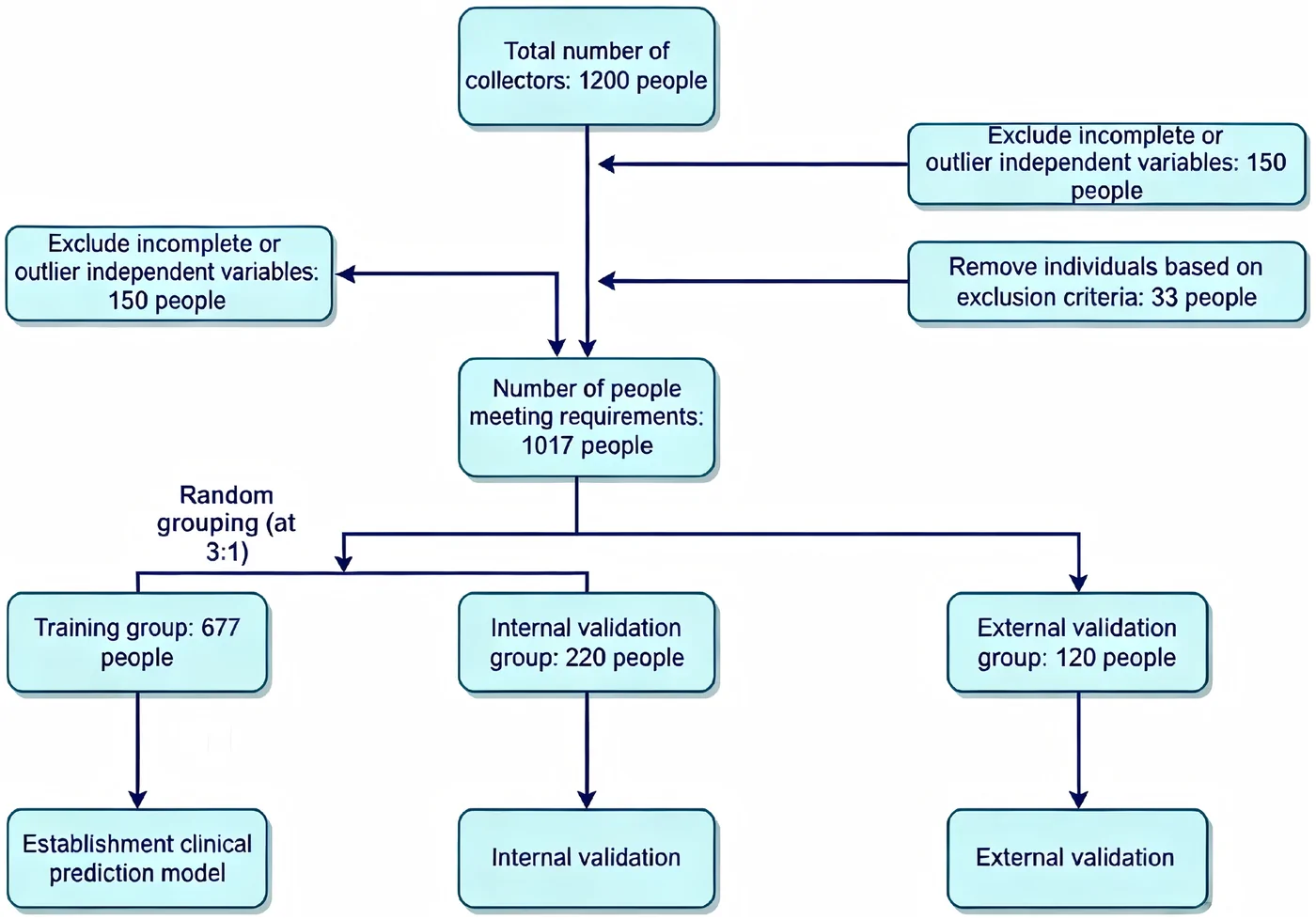
Disposition of patients. Eligible patients were divided into three groups: training group, internal validation group, and external validation group.

The median Kt/V values were 1.33 (Q1–Q3: 1.14–1.56) in the training set, 1.31 (Q1–Q3: 1.11–1.51) in the internal validation set, and 1.31 (Q1–Q3: 1.11–1.51) in the external validation set. The training set had a median age of 55.0 years (Q1–Q3: 44.0–65.0), with 396 (58.5%) male patients. The median dialysis vintage was 42.0 months (Q1–Q3: 15.0–87.8). The mean body surface area (BSA) was 1.81 ± 0.19 m^2^ in the training set. Detailed baseline characteristics of the three datasets are presented in Table 1.

**Table 1:** Baseline characteristics of participants.

Characteristic	Total (*n* = 1,017)	Training (*n* = 677)	Internal validation (*n* = 220)	External validation (*n* = 120)
Kt/V	1.34 (1.14, 1.55)	1.33 (1.14, 1.56)	1.31 (1.11, 1.51)	1.31 (1.11, 1.51)
Sex				
Male	600 (59.00)	396 (58.50)	127 (57.73)	77 (64.17)
Female	417 (41.00)	281 (41.50)	93 (42.27)	43 (35.83)
Ethnicity				
Han	908 (89.28)	611 (90.25)	191 (86.82)	106 (88.33)
Mongolian	81 (7.97)	51 (7.53)	20 (9.09)	10 (8.34)
Other	28 (2.75)	15 (2.22)	9 (4.09)	4 (3.33)
Age, years	55.00 (44.00, 65.00)	56.00 (46.00, 65.00)	55.00 (44.00, 65.00)	55.00 (44.00, 65.00)*
Primary disease				
Diabetes mellitus	172 (16.91)	122 (18.02)	34 (15.45)	16 (13.33)
Chronic glomerulonephritis	104 (10.22)	76 (11.23)	18 (8.18)	10 (8.33)
Hypertension	267 (26.25)	168 (24.82)	50 (22.73)	49 (40.84)
Tubulointerstitial disease	31 (3.05)	20 (2.95)	8 (3.64)	3 (2.50)
Autoimmune disease	33 (3.25)	20 (2.95)	13 (5.91)	0 (0)
Polycystic kidney disease	33 (3.25)	17 (2.51)	13 (5.91)	3 (2.50)
Primary nephrotic syndrome (pathology unknown)	19 (1.87)	12 (1.77)	6 (2.73)	1 (0.83)
IgA nephropathy	20 (1.97)	15 (2.22)	4 (1.82)	1 (0.83)
Drug-induced injury	128 (12.58)	96 (14.18)	21 (9.54)	11 (9.17)
Unexplained reasons	210 (20.65)	131 (19.35)	53 (24.09)	26 (21.67)
Dialysis frequency				
2/week	66 (6.49)	35 (5.17)	24 (10.91)	8 (6.70)
2.5/week	146 (14.36)	105 (15.51)	28 (12.73)	13 (10.80)
3 or more/week	805 (79.15)	537 (79.32)	168 (76.36)	99 (82.50)
Dialysis duration, months	39.50 (14.00, 86.00)	42.00 (15.00, 87.75)	38.00 (12.00, 84.00)	18.00 (12.00, 36.00)
BSA, m^2^	1.82 ± 0.19	1.81 ± 0.19	1.85 ± 0.22	1.81 ± 0.18
Arteriovenous fistula or hemodialysis catheter usage duration, months	34.00 (12.00, 69.00)	35.00 (12.00, 69.75)	32.00 (9.00, 69.00)	12.00 (12.00, 36.00)
Dialysate sodium ion concentration, mmol/L	138.00 (135.00, 140.00)	139.00 (135.00, 140.00)	138.00 (135.00, 140.00)	138.00 (136.00, 140.00)*
Dialysate flow rate, mL/min	500 (500, 500)	500 (500, 500)	500 (500, 500)	500 (500, 500)
Pre-dialysis weight, kg	64.00 (55.25, 73.10)	63.7 (54.95, 72.90)	65.30 (56.73, 74.35)	65.30 (56.73, 74.4)
Post-dialysis weight, kg	62.40 (53.55, 71.45)	62.60 (53.63, 71.55)	65.20 (55.20, 73.40)	52.50 (48.80, 58.00)*
Dialyzer membrane area, m^2^	1.80 (1.40, 1.80)	1.80 (1.40, 1.80)	1.80 (1.40, 1.80)	1.80 (1.40, 1.80)*
Dialyzer material				
Polysulfone membrane	456 (44.83)	307 (45.35)	105 (47.73)	44 (36.67)*
Polysulfone fiber	201 (19.76)	149 (22.01)	48 (21.82)	4 (3.33)*
Cellulose membrane	95 (9.34)	41 (6.06)	21 (9.54)	33 (27.50)*
PVP mixtures	110 (10.82)	72 (10.63)	15 (6.82)	23 (19.17)*
Polyamides	83 (8.16)	55 (8.12)	12 (5.45)	16 (13.33)*
Polyethersulfone membrane	72 (7.09)	53 (7.83)	19 (8.64)	0 (0.00)
Actual length of dialysis, h	4.00 (4.00, 4.00)	4.00 (4.00, 4.00)	4.00 (4.00, 4.00)	4.00 (4.00, 4.00)
Actual ultrafiltration volume, L	2.50 (1.80, 3.30)	2.50 (1.80, 3.20)	2.30 (1.60, 3.18)	2.30 (1.60, 3.18)
Anticoagulant type				
Low-molecular heparin	960 (94.40)	637 (94.09)	207 (94.09)	116 (96.67)
Ordinary heparin	36 (3.54)	27 (3.99)	8 (3.64)	1 (0.83)
No heparin	21 (2.06)	13 (1.92)	5 (2.27)	3 (2.50)
Actual blood flow, mL/min	230.00 (220.00, 240.00)	230.00 (220.00, 240.00)	230.00 (210.00, 230.00)	230.00 (210.00, 230.00)
Access type				
Arteriovenous fistula	807 (79.35)	537 (79.32)	169 (76.82)	101 (84.17)
Temporary dialysis catheter	63 (6.20)	40 (5.91)	19 (8.64)	4 (3.33)
Long-term dialysis catheter	147 (14.45)	100 (14.77)	32 (14.54)	15 (12.50)
Albumin, g/L	37.00 (35.00, 39.00)	37.00 (35.00, 39.00)	37.00 (35.00, 39.00)	37.00 (35.00, 39.00)
Total cholesterol, mmol/L	3.95 (3.29, 4.65)	3.88 (3.29, 4.63)	4.27 (3.50, 4.80)	4.27 (3.50, 4.76)
Triglycerides, mmol/L	1.70 (1.14, 2.51)	1.68 (1.13, 2.46)	1.88 (1.16, 2.90)	1.88 (1.16, 2.90)
Hemoglobin, g/L	105.14 ± 17.82	105.12 ± 17.51	105.50 ± 17.65	104.91 ± 18.70
C-reactive protein, mg/dL	4.03 (1.49, 11.09)	3.99 (150, 12.39)	4.12 (1.53, 10.07)	2.25 (0.70, 5.01)*
Platelets, ×10^9^/L	164.50 (135.00, 211.00)	164.00 (134.00, 209.00)	173.00 (142.00, 214.00)	171.00 (135.00, 230.00)*
PTH, pg/mL	234.65 (134.77, 409.37)	229.20 (130.60, 406.40)	250.60 (152.40, 384.40)	398.10 (182.30, 545.00)*
Serum phosphorus, mmol/L	1.85 (1.51, 2.25)	1.83 (1.50, 2.24)	1.94 (1.50, 2.27)	1.94 (1.69, 2.35)*
Serum calcium, mmol/L	2.18 (2.02, 2.35)	2.18 (2.02, 2.36)	2.19 (2.02, 2.38)	2.07 (1.95, 2.24)*
Serum potassium, mmol/L	4.98 (4.39, 5.60)	4.99 (4.40, 5.65)	4.84 (4.33, 5.60)	5.08 (4.96, 5.29)*

Normally distributed continuous data are presented as mean ± standard deviation, non-normally distributed continuous data as median (Q1, Q3), and categorical data as *n* (%).

* *P* < 0.05 for comparison with the training set.

### 3.2 Feature engineering and selection

From the original 26 candidate variables, we constructed eight clinically meaningful derived features: circulating blood volume, standardized blood flow, ultrafiltration rate, pulse pressure, mean arterial pressure, lipid accumulation index, nutritional index, and weight to BSA ratio.

Using a multi-algorithm fusion strategy that integrated rankings from seven different feature selection methods (*F* test, Mutual Information, Random Forest importance, XGBoost importance, LASSO coefficients, Permutation Importance and Recursive Feature Elimination), we generated a consensus importance ranking of all candidate features. Besides, the Boruta algorithm provided the importance classification for each candidate variable (confirmed, rejected, or tentative), based on its importance relative to shadow features. Table 2 shows the ranking of feature selection for the top ten features and the ranking of feature selection for all the features are shown in Supplementary Table S2. Based on the average rank and clinical expert review, five features were selected as the final input variables for model development: (1) Body surface area (BSA, m^2^); (2) Pre-dialysis weight (kg); (3) Standardized blood flow (mL/min/m^2^); (4) Sex; (5) Circulating blood volume (mL).

**Table 2:** Feature selection ranking across eight algorithms for the top ten features.

Feature	F ranking	MI ranking	RF ranking	XGB ranking	LASSO ranking	Perm ranking	RFE ranking	Average ranking	Top 5 times	Boruta grade	Boruta status
Body surface area	1	2	1	2	1	1	1	1.285714	7	1	Confirmed
Pre-dialysis weight	2	1	3	3	13	4	2	4	6	1	Confirmed
Standardized blood flow	5	3	2	1	13	2	6	4.571429	5	1	Confirmed
Weight-to-BSA ratio	3	4	6	12	10	6	8	7	2	3	Rejected
Circulating blood volume	6	8	10	8	3	10	5	7.142857	2	4	Rejected
Sex	4	5	18	5	2	7	11	7.428571	4	8	Rejected
Total cholesterol	8	9	5	15	7	5	13	8.857143	2	2	Tentative
Lipid accumulation index	16	26	4	7	13	3	12	11.57143	2	1	Confirmed
Actual dialysis duration	10	12	19	4	6	21	19	13	1	17	Rejected
Actual ultrafiltration volume	13	13	20	17	8	20	3	13.42857	1	17	Rejected

F, *F* test; MI, Mutual information; RF, Random forest importance; XGB, XGBoost importance; LASSO, LASSO coefficients; Perm, permutation importance; RFE, recursive feature elimination.

The feature “weight-to-BSA ratio” was excluded due to high collinearity with BSA and pre-dialysis weight, and because its importance rank was lower than that of the selected variables.

For the multiple linear regression (MLR) model, the final fitted Equation 5 was

$$\begin{array}{rl} Kt/V & =1.305-0.276\times BSA(m^{2})+0.010\times predialysis\;weight(kg)-0.240\\ & \times standardized\;blood\;flow\;(ml/min)+\;0.110\\ & \times sex(1\;for\;female,0\;for\;male)+0.276\\ & \times circulating\;blood\;volume(ml) \end{array}$$ (5)

### 3.3 Hyperparameter tuning outcomes

Hyperparameter optimization was performed using GridSearchCV with 5-fold cross validation on the training set, with the objective of maximizing the average R^2^ across folds. The optimal hyperparameters for each model are summarized in Table 3. The full hyperparameters search spaces are provided in Supplementary Table S1.

**Table 3:** The optimal hyperparameters for each model.

Model	The optimal hyperparameters
XGBoost	n_estimators=100, max_depth=5, learning_rate=0.05, subsample=0.8, colsample_bytree=0.8, gamma=0.1, reg_lambda=1.0, min_child_weight=1
LightGBM	n_estimators=200, num_leaves=15, learning_rate=0.01, subsample=0.8, colsample_bytree=0.8, reg_alpha=0, reg_lambda=0.01
RandomForest	n_estimators=100, max_depth=5, max_features=’sqrt’, min_samples_leaf=2, min_samples_split=5
GradientBoosting	n_estimators=300, learning_rate=0.01, max_depth=3, subsample=0.7, min_samples_split=10, min_samples_leaf=1
KNN	n_neighbors=9, weights='uniform’, p=2
SVM	C=50, gamma=0.01, epsilon=0.2, kernel='rbf'
BPNN	hidden_layer_sizes=(5,3), activation='tanh’, alpha=0.05, learning_rate_init=0.005
MLR	Not applicable

For XGBoost, the optimal configuration was n_estimators=100, max_depth=5, learning_rate=0.05, subsample=0.8, colsample_bytree=0.8, gamma=0.1, reg_lambda=1.0, and min_child_weight=1. For SVM with RBF kernel, the best parameters were C=50, gamma=0.01, and epsilon=0.2. For the BPNN, the optimal architecture consisted of two hidden layers with 5 and 3 nodes respectively (hidden_layer_sizes=(5,3)), activation='tanh', alpha=0.05, and learning_rate_init=0.005. For KNN, the optimal n_neighbors was 9 with weights='uniform' and p=2.

### 3.4 Model performance and model selection

#### 3.4.1 Model performance on internal validation

All eight individual models and the ensemble model were evaluated on the internal validation set (*n* = 220), which was strictly withheld from all training and tuning procedures. Performance metrics on internal validation are presented in Table 4.

**Table 4:** Performance metrics of point prediction on internal and external validation sets.

Model	Val_R^2^	Val_RMSE	Val_MAE	Test_R^2^	Test_RMSE	Test_MAE
MLR	0.3337	0.2516	0.1574	0.5389	0.1739	0.1278
XGBoost	0.4293	0.2329	0.1628	0.5681	0.1683	0.1288
LightGBM	0.4155	0.2357	0.1657	0.5562	0.1706	0.1289
RandomForest	0.3873	0.2413	0.1703	0.5586	0.1701	0.1324
GradientBoosting	0.4126	0.2362	0.1648	0.5748	0.167	0.1276
KNN	0.4003	0.2387	0.1684	0.5135	0.1786	0.1374
SVM	0.4633	0.2258	0.1512	0.5931	0.1633	0.1214
BPNN	0.4197	0.2348	0.163	0.5407	0.1735	0.1287
Ensemble (Avg)	0.4478	0.2291	0.1582	0.5911	0.1637	0.124

Val refers to internal validation set and Test refers to external validation set. Ensemble (Avg) refers to a simple average ensemble combining the predictions of the top performing models (SVM, GradientBoosting, and XGBoost), which are selected based on their superior test set RMSE and MAE.

On the internal validation set, the SVM model achieved the highest R^2^ (0.4633), the lowest RMSE (0.2258), and the lowest MAE (0.1512) among individual models. The GradientBoosting model achieved an R^2^ of 0.4126, RMSE of 0.2362, and MAE of 0.1648. The XGBoost model achieved an R^2^ of 0.4293, RMSE of 0.2329, and MAE of 0.1628. The MLR baseline model achieved an R^2^ of 0.3337, RMSE of 0.2516, and MAE of 0.1574.

The simple average ensemble (combining SVM, GradientBoosting, and XGBoost) outperformed all individual models on the internal validation set, achieving an R^2^ of 0.4478, RMSE of 0.2291, and MAE of 0.1582. The BPNN, LightGBM, RandomForest, and KNN models demonstrated intermediate performance, with R^2^ values ranging from 0.3873 to 0.4197 on the internal validation set.

#### 3.4.2 Model performance on external validation

To assess generalizability to completely unseen patient populations from different clinical centers, all models were evaluated on the external validation set (*n* = 120). Performance metrics on external validation are presented in Table 4. On the external validation set, all models demonstrated improved performance compared to the internal validation set, which may be attributed to the smaller standard deviation of Kt/V in the external validation cohort (0.257 vs. 0.309 in the internal validation set).

The SVM model continued to perform best among individual models, achieving an R^2^ of 0.5931, RMSE of 0.1633, and MAE of 0.1214. The GradientBoosting model achieved an R^2^ of 0.5748, RMSE of 0.1670, and MAE of 0.1276. The XGBoost model achieved an R^2^ of 0.5681, RMSE of 0.1683, and MAE of 0.1288.

The simple average ensemble achieved the highest overall performance on the external validation set, with an R^2^ of 0.5911, RMSE of 0.1637, and MAE of 0.1240, demonstrating robust generalizability across different clinical settings. Notably, the MLR baseline model also performed competitively on the external validation set (R^2^=0.5389, RMSE=0.1739, MAE=0.1278), suggesting that the selected linear features capture substantial predictive information.

#### 3.4.3 Model selection for clinical translation

To select the most suitable models for clinical implementation, we ranked all individual models by their RMSE and MAE on the external validation set. The SVM, GradientBoosting, and XGBoost models consistently ranked among the top three on both metrics. These three algorithms were therefore chosen as the final model set for subsequent application development, as they demonstrated the best trade-off between predictive accuracy and generalizability across different clinical centers. Moreover, their simple average ensemble achieved even higher performance on the external validation set (R^2^ = 0.5911, RMSE = 0.1637), and was thus also incorporated into the final application interface, alongside the three individual models.

### 3.5 Calibration assessment

To determine whether the predictions from the three selected individual models and their ensemble exhibited systematic bias, we performed a calibration diagnosis on the internal validation set. The calibration parameters, including the slope (a), intercept (b), residual mean, residual standard deviation, MAE improvement after calibration, and *Δ*R^2^, for each model are summarized in Table 5.

**Table 5:** Calibration judgment results of each model.

Model	Slope (a)	Intercept (b)	Residual mean	Residual standard deviation	MAE improvement	*Δ*R^2^	Suggested calibration
SVM	0.9333	0.0958	0.0028	0.1633	0.0004	0.0032	No
GradientBoosting	0.9832	0.0294	0.0059	0.1654	0.0006	0.0007	No
XGBoost	1.0473	−0.0499	0.0155	0.166	0.0015	0.0049	No
Ensemble (Avg)	1.0038	0.0029	0.0081	0.1624	0.0003	0.001	No

For the SVM model, the calibration slope was 0.9333 and the intercept was 0.0958, with a residual mean of 0.0028 and a residual standard deviation of 0.1633. For the GradientBoosting model, the slope was 0.9832 and the intercept was 0.0294, with a residual mean of 0.0059 and a residual standard deviation of 0.1654. For the XGBoost model, the slope was 1.0473 and the intercept was −0.0499, with a residual mean of 0.0155 and a residual standard deviation of 0.1660. For the simple average ensemble, the calibration slope was 1.0038 and the intercept was 0.0029, with a residual mean of 0.0081 and a residual standard deviation of 0.1624.

The residuals for all four models were symmetrically distributed around zero with approximately normal distributions, as confirmed by the residual-vs.-predicted scatter plots, histograms, true-vs.-predicted scatter plots, and Q-Q plots (Figure 2 for the ensemble; Supplementary Figures S1–S3 for the three individual models). The true-vs.-predicted scatter plots showed points closely aligned with the 45° diagonal line, indicating no substantial systematic over- or under-estimation.

**Figure 2:**
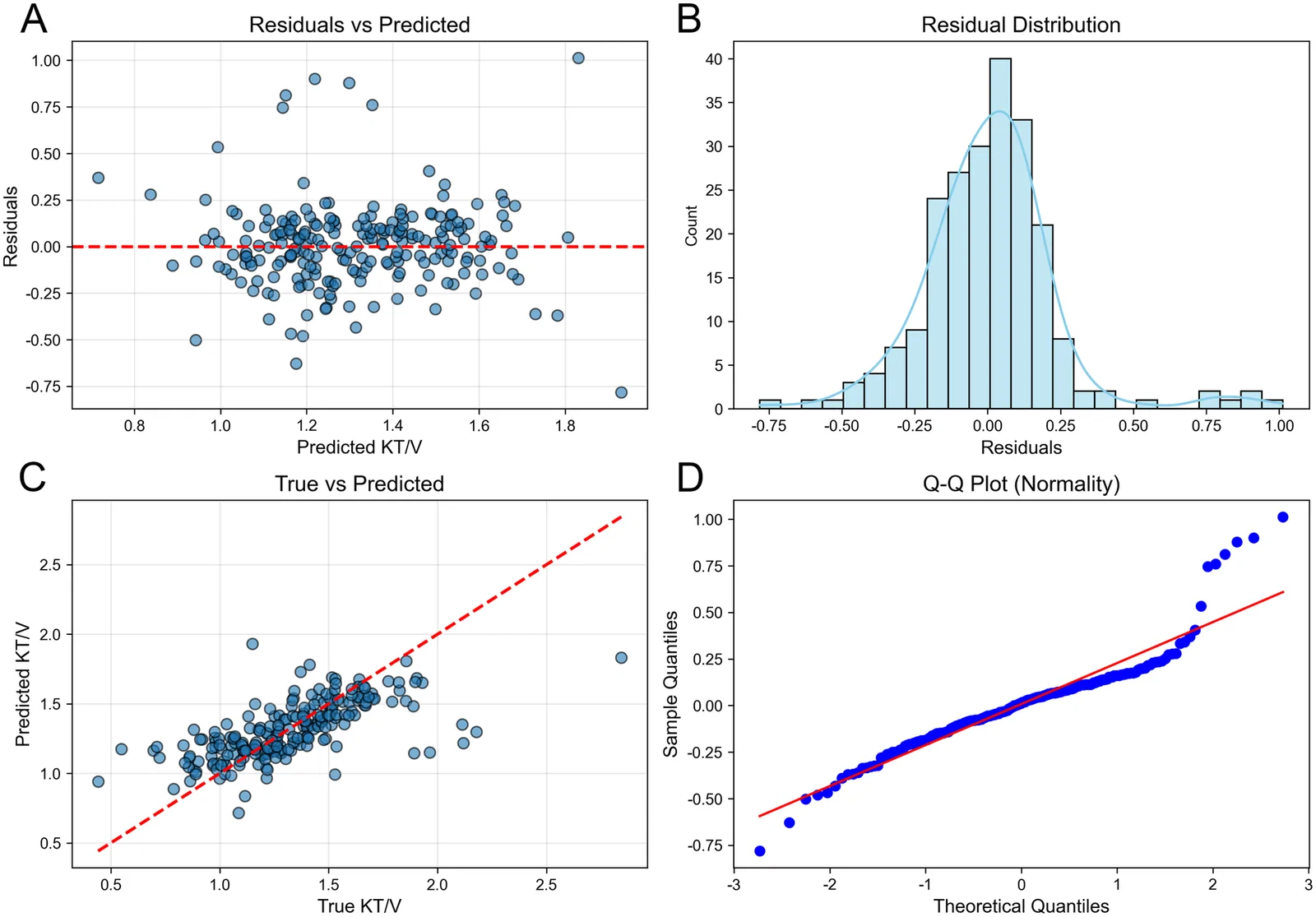
Calibration assessment on the ensemble of three selected models. **(A)** Rresidual-vs.-predicted plots; **(B)** histogram; **(C)** true-vs.-predicted scatter plots; **(D)** Q-Q plots.

Consistent with these findings, *post-hoc* linear calibration resulted in only marginal improvements in MAE across all models: 0.0004 for SVM, 0.0006 for GradientBoosting, 0.0015 for XGBoost, and 0.0003 for the ensemble, all well below the pre-defined threshold of 0.005. The changes in R^2^ after calibration were also negligible (+0.0032, +0.0007, +0.0049, and +0.0010, respectively). The negligible improvements confirmed that all four models were already well calibrated and did not require additional post-processing. The ensemble model, with its calibration slope closest to 1 and intercept closest to 0 among all models, demonstrated the best overall calibration and was therefore selected as the final model for clinical application.

### 3.6 Feature importance and model interpretability

To elucidate the contribution of each feature to the model predictions, we performed SHAP (SHapley Additive exPlanations) analysis for the SVM and GradientBoosting models (Figures 3,4).

**Figure 3:**
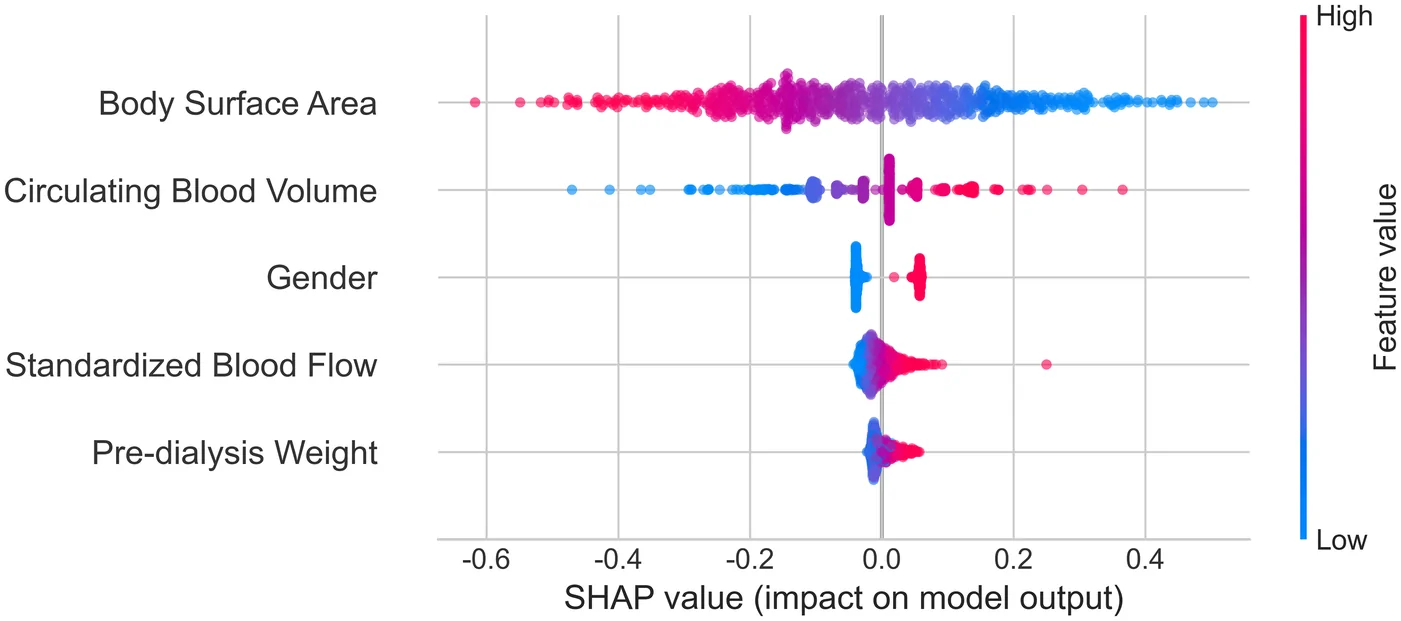
SHAP beeswarm plot for the SVM model.

**Figure 4:**
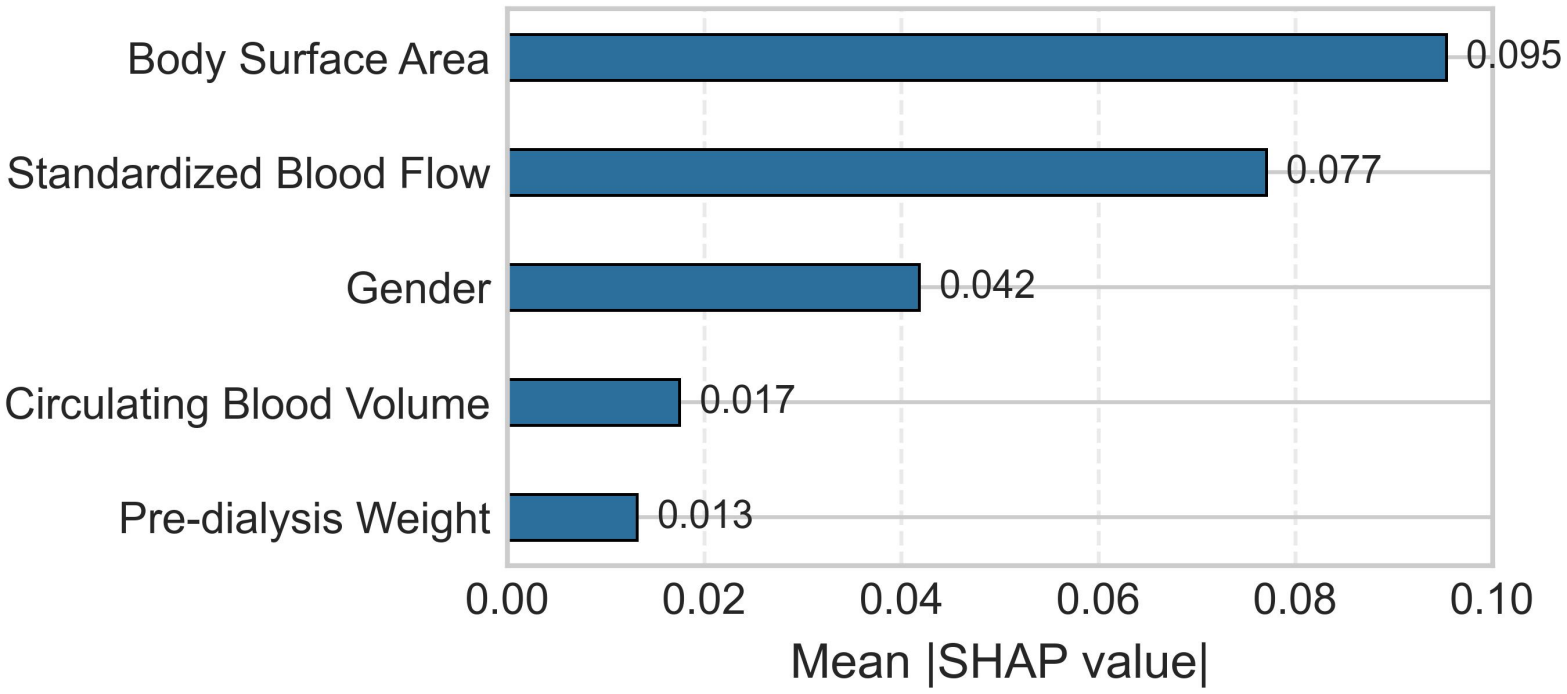
SHAP summary bar plot for the GradientBoosting model.

For the SVM model, the SHAP beeswarm plot revealed that BSA was the most influential feature, followed by circulating blood volume, gender, standardized blood flow, and pre-dialysis weight. Higher BSA values were associated with negative SHAP values (i.e., lower Kt/V predictions), consistent with the clinical expectation that larger body size requires higher dialysis doses to achieve adequate clearance. For the GradientBoosting model, the SHAP summary bar plot showed a similar ranking: BSA had the highest mean SHAP value (0.095), followed by standardized blood flow (0.077), gender (0.042), circulating blood volume (0.017), and pre-dialysis weight (0.013).

These consistent rankings across fundamentally different model architectures (geometric SVM vs*.* tree based GradientBoosting) reinforce the robustness of the feature importance findings.

### 3.7 Probabilistic prediction performance

To quantify prediction uncertainty, we evaluated two probabilistic prediction methods: LightGBM quantile regression and NGBoost. Performance metrics of probabilistic prediction on internal and external validation sets are presented in Table 6.

**Table 6:** Performance metrics of probabilistic prediction on internal and external validation sets.

Model	Data set	PICP_80	MPIW	CWC	CRPS	NLL
LightGBM Quantile	Validation set	86.82%	0.6123	0.6123	–	–
NGBoost	Validation set	82.27%	0.4924	0.4924	0.124	0.0084
LightGBM Quantile	Test set	92.50%	0.5826	0.5826	–	–
NGBoost	Test set	88.33%	0.4838	0.4838	0.0936	−0.3701

Validation set refers to internal validation set, and Test set refers to external validation set. CRPS and NLL are not applicable to quantile regression models.

On internal validation, for the 80% prediction interval, NGBoost achieved a PICP (Prediction Interval Coverage Probability) of 82.27%, which closely approximates the nominal 80% coverage, with an MPIW (Mean Prediction Interval Width) of 0.4924 and a CWC (Coverage Width Criterion) of 0.4924. The CRPS (Continuous Ranked Probability Score) was 0.1240, and the NLL (Negative Log Likelihood) was 0.0084 on internal validation.

On internal validation, LightGBM quantile regression achieved a PICP of 86.82% (overly conservative), with an MPIW of 0.6123 and a CWC of 0.6123. While LightGBM covered more true values on internal validation, this came at the cost of substantially wider prediction intervals (approximately 24% wider than NGBoost).

Based on its superior calibration (PICP closer to nominal 80%), narrower prediction intervals, and ability to provide complete probability density information on internal validation, NGBoost was selected as the preferred method for clinical uncertainty quantification.

### 3.8 Clinical application interface

To facilitate clinical translation, we packaged the final ensemble model and the NGBoost probabilistic predictor into a local standalone executable application. This application is distributed as a self-contained file package that can be copied directly to a local computer and executed offline, without requiring any network connection.

The interface accepts five input parameters (sex, height, pre-dialysis weight, blood flow rate, and dialysis duration) and returns: (1) Individual model point predictions (SVM, GradientBoosting and XGBoost); (2) Ensemble average prediction; (3) NGBoost derived median and 80% prediction interval; (4) Probability density curve visualization.

A screenshot of the application interface is provided in Figure 5. The complete executable package and source code are available from the corresponding author upon reasonable request.

**Figure 5:**
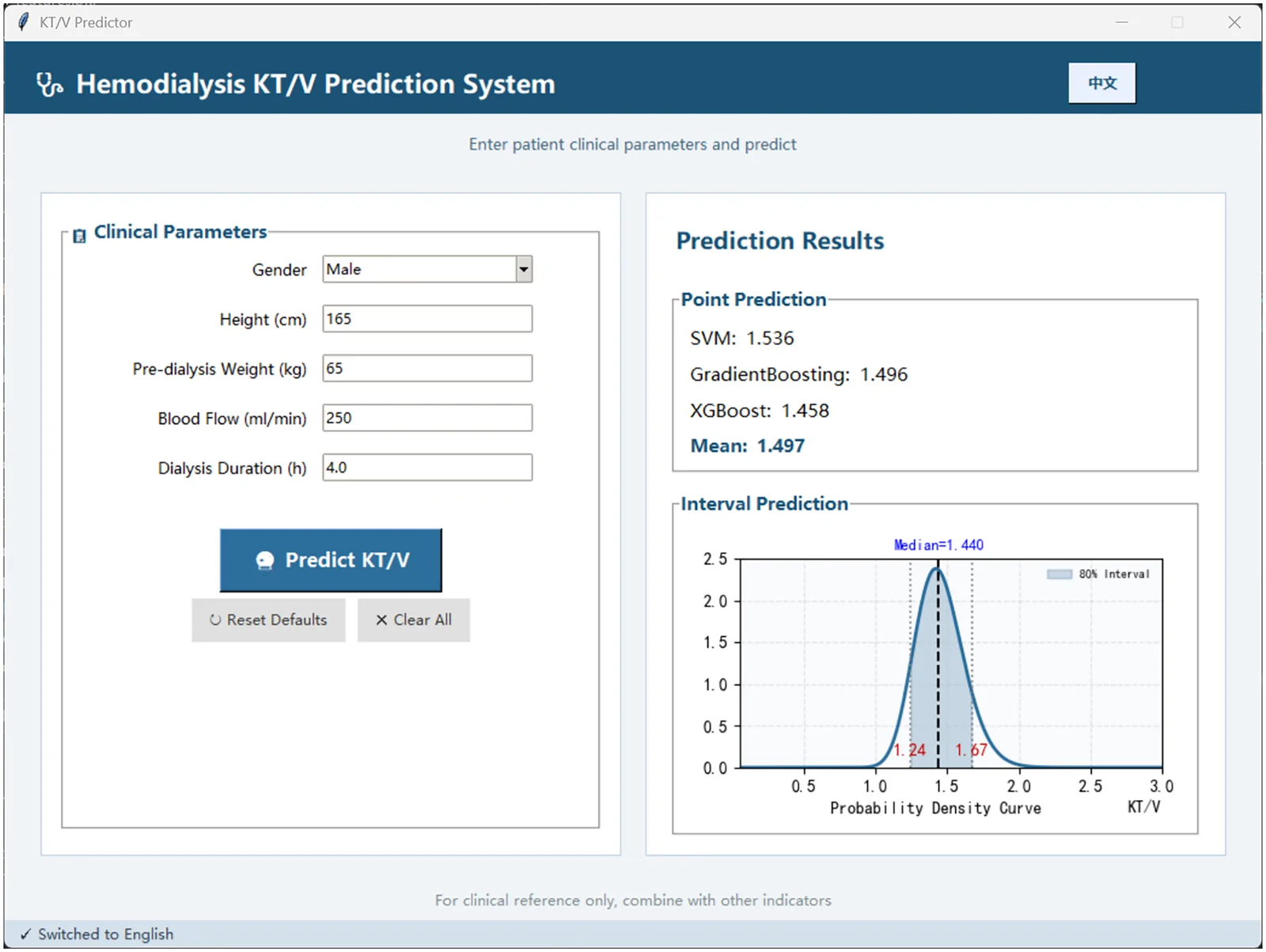
Screenshot of the application interface.

## 4 Discussion

### 4.1 Study overview

This multicenter study developed and validated predictive models for hemodialysis adequacy (Kt/V) using multiple linear regression, seven machine learning algorithms for point prediction, an ensemble model, and two probabilistic prediction methods. The key findings are: (1) the SVM model consistently outperformed other individual models across both internal and external validation sets, achieving an R^2^ of 0.5931 and RMSE of 0.1633 on external validation; (2) the simple average ensemble of SVM, GradientBoosting, and XGBoost achieved the highest overall performance (R^2^ = 0.5911, RMSE = 0.1637); (3) NGBoost provided well-calibrated prediction intervals (PICP of 82.27% for nominal 80% coverage) with narrower intervals than quantile regression; and (4) BSA, standardized blood flow, and gender were consistently identified as the most influential predictors across different modeling frameworks. This study was strictly designed, implemented and reported according to the TRIPOD + AI guidelines for machine learning-based prediction models (14).

### 4.2 Methodological contributions and innovations

From a methodological perspective, this study makes several contributions to the field of clinical prediction modeling for continuous outcomes. First, we employed a multi-algorithm fusion strategy for feature selection, integrating eight fundamentally different methods (*F* test, Mutual Information, Random Forest importance, XGBoost importance, LASSO coefficients, Permutation Importance, Recursive Feature Elimination, and Boruta) to generate a consensus importance ranking. The Boruta algorithm, a random forest-based wrapper method that iteratively compares feature importance with randomly permuted “shadow” features, has been shown to offer high stability and effectiveness in high-dimensional clinical datasets. By averaging rankings across multiple algorithms, our approach reduces the risk of selecting features that are important only under a single methodological paradigm. This ensemble-based selection strategy represents a more robust alternative to the conventional stepwise regression approach, which has been criticized for its instability and tendency to capitalize on chance.

Second, we addressed the critical issue of prediction uncertainty quantification, a dimension often neglected in clinical prediction model studies. For continuous outcomes such as Kt/V, providing only a point estimate without corresponding uncertainty information limits the clinical utility of the model, as clinicians cannot gauge the reliability of individual predictions. We implemented NGBoost (Natural Gradient Boosting) (15), an algorithm that generalizes gradient boosting to probabilistic regression by treating the parameters of the conditional distribution as targets for a multiparameter boosting algorithm. Unlike conventional regression models that return only a point estimate, NGBoost outputs a full probability distribution over the outcome space, enabling predictive uncertainty estimation. The Natural Gradient correction ensures stable training dynamics for the multiparameter approach. In our validation, NGBoost achieved a PICP of 82.27% for the nominal 80% prediction interval, with an MPIW of 0.4924, substantially narrower than the 0.6123 width of LightGBM quantile regression. This superior calibration (narrowness trade-off) suggests that NGBoost provides clinically meaningful uncertainty estimates without being overly conservative. These findings are consistent with previous reports demonstrating NGBoost's competitive performance in probabilistic prediction tasks across various domains.

Third, we enhanced model interpretability using SHAP (SHapley Additive exPlanations) analysis. SHAP, a model agnostic framework based on coalition game theory (16), quantifies the marginal contribution of each feature to individual predictions. The consistent ranking of BSA as the most influential predictor across both the SVM (geometric) and GradientBoosting (tree-based) models reinforces the robustness of this finding. SHAP analysis has been increasingly recognized as a valuable tool for translating “black box” machine learning models into clinically actionable insights, facilitating clinician trust and fair model evaluation. By providing both global feature importance rankings and local explanations for individual predictions, our approach addresses the interpretability requirements emphasized in the TRIPOD + AI guidelines.

### 4.3 Clinical translation and practical utility

A key contribution of this study is the explicit bridging of *post-hoc* model development with pre-dialysis clinical application. Although the model was developed using actual post-dialysis parameters (the only objectively documented values available in the retrospective dataset), its intended clinical use is pre-dialysis prediction. In routine practice, dialysis prescriptions, including planned duration, blood flow rate, and ultrafiltration volume, are determined by clinicians before the session begins. The high concordance between prescribed and delivered parameters (mean absolute difference <2% in our dataset) ensures that planned values can be substituted into the model to obtain reliable predictions of expected Kt/V. This enables real-time, pre-emptive adjustment of dialysis prescriptions, allowing clinicians to modify parameters before the session starts if the predicted Kt/V falls below target.

This functionality fundamentally distinguishes our approach from the Daugirdas formula, which requires post-dialysis urea measurements and therefore can only provide retrospective adequacy assessment. Our model offers prospective decision support, potentially reducing the proportion of sessions with inadequate dialysis and its associated complications.

The features selected by our consensus ranking strategy, BSA, pre-dialysis weight, standardized blood flow, sex, and circulating blood volume, are all non-invasive and readily available at the point of care. This is consistent with recent findings that machine learning models based on non-invasively collected general and dialysis-related variables can maintain high prediction accuracy. The identification of vascular access, gender, BMI, ultrafiltration volume, and dialysis duration as significant predictors in previous studies further supports the clinical plausibility of our selected features.

### 4.4 Comparison with previous studies

Previous attempts to predict Kt/V using machine learning have been limited by single center designs, lack of external validation, and insufficient methodological detail. Alexander et al. (2003) used MLR, tree-based modeling, and artificial neural networks but achieved a correlation coefficient of only 0.745 and lacked internal and external validation (17). Barbieri C et al. (2019) developed a multi-endpoint ANN model but relied on a single center and did not describe predictive performance or conduct feature importance analysis (18). More recent studies have demonstrated the feasibility of machine learning for dialysis adequacy prediction using demographic, intradialytic, and laboratory variables (19). He et al. proposed a domain-adversarial neural network for Kt/V prediction (20), achieving high accuracy on data from 17 patients. However, these studies predominantly focused on point prediction without addressing uncertainty quantification or model interpretability.

Our study addresses these gaps through: (1) a large multicenter sample (1,017 patients from seven centers); (2) comprehensive internal and external validation; (3) probabilistic prediction with well-calibrated uncertainty intervals; (4) multi algorithm feature selection with consensus ranking; (5) SHAP-based interpretability; and (6) a deployable local standalone application for clinical use.

## 5 Limitations

Several limitations should be acknowledged. First, the training set size (*n* = 677) and external validation set (*n* = 120) are moderate. Although our sample size exceeds the “10 events per predictor” rule of thumb for the five selected features (21), future studies with larger and more diverse populations are needed to confirm generalizability. Second, the study population was drawn from a single geographic region (Inner Mongolia, China), which may limit applicability to other ethnic groups and healthcare settings. Third, while we demonstrated excellent performance on internal and external validation, prospective deployment studies are required to evaluate real-world clinical impact. Fourth, the NGBoost probabilistic predictions assume a log-normal distribution for log transformed Kt/V; although our residual diagnostics supported this assumption, future work could explore distribution-free uncertainty quantification methods such as conformal prediction. Fifth, the simple average ensemble, while effective, could potentially be improved through more sophisticated stacking or Bayesian model averaging approaches.

## 6 Recommendations

Future research should focus on: (1) prospective validation of the model in routine clinical practice, ideally with randomized controlled trial designs to evaluate impact on patient outcomes; (2) expansion to diverse geographic and ethnic populations; (3) integration of the prediction tool into electronic health record systems for seamless clinical workflow integration; (4) exploration of deep learning approaches that can capture complex temporal patterns in dialysis data; and (5) investigation of model fairness across different demographic subgroups, as recommended by the TRIPOD + AI framework.

## 7 Conclusion

This study demonstrates that machine learning models, particularly SVM, ensemble averaging, and NGBoost, can provide accurate, interpretable, and uncertainty-aware predictions of hemodialysis adequacy (Kt/V) using non-invasive, readily available parameters. The methodological innovations, including multi-algorithm feature selection, probabilistic prediction with NGBoost, and SHAP-based interpretability, collectively address the key limitations identified in previous studies and align with the updated TRIPOD + AI reporting standards. The development of a local standalone executable application further facilitates clinical translation, enabling pre-dialysis prediction and real-time prescription adjustment. If validated prospectively, this approach has the potential to improve dialysis quality, reduce complications, and support personalized treatment planning for patients receiving maintenance hemodialysis.

## Data Availability

The original contributions presented in the study are included in the article/Supplementary Material, further inquiries can be directed to the corresponding authors.
