# Supplementary material for: Development and validation of predictive models for the hemodialysis adequacy index (Kt/V) using multiple linear regression and machine learning algorithms
Source: Front Digit Health. 2026 Oct 12;8:1771806. doi: 10.3389/fdgth.2026.1771806 (PMC13699731; doi:10.3389/fdgth.2026.1771806)
Supplement: Supplementary file 1 [file Supplementaryfile1.pdf]

Supplementary Figures S1-3. Calibration assessments on the XGBoost, SVM and GradientBoosting models.

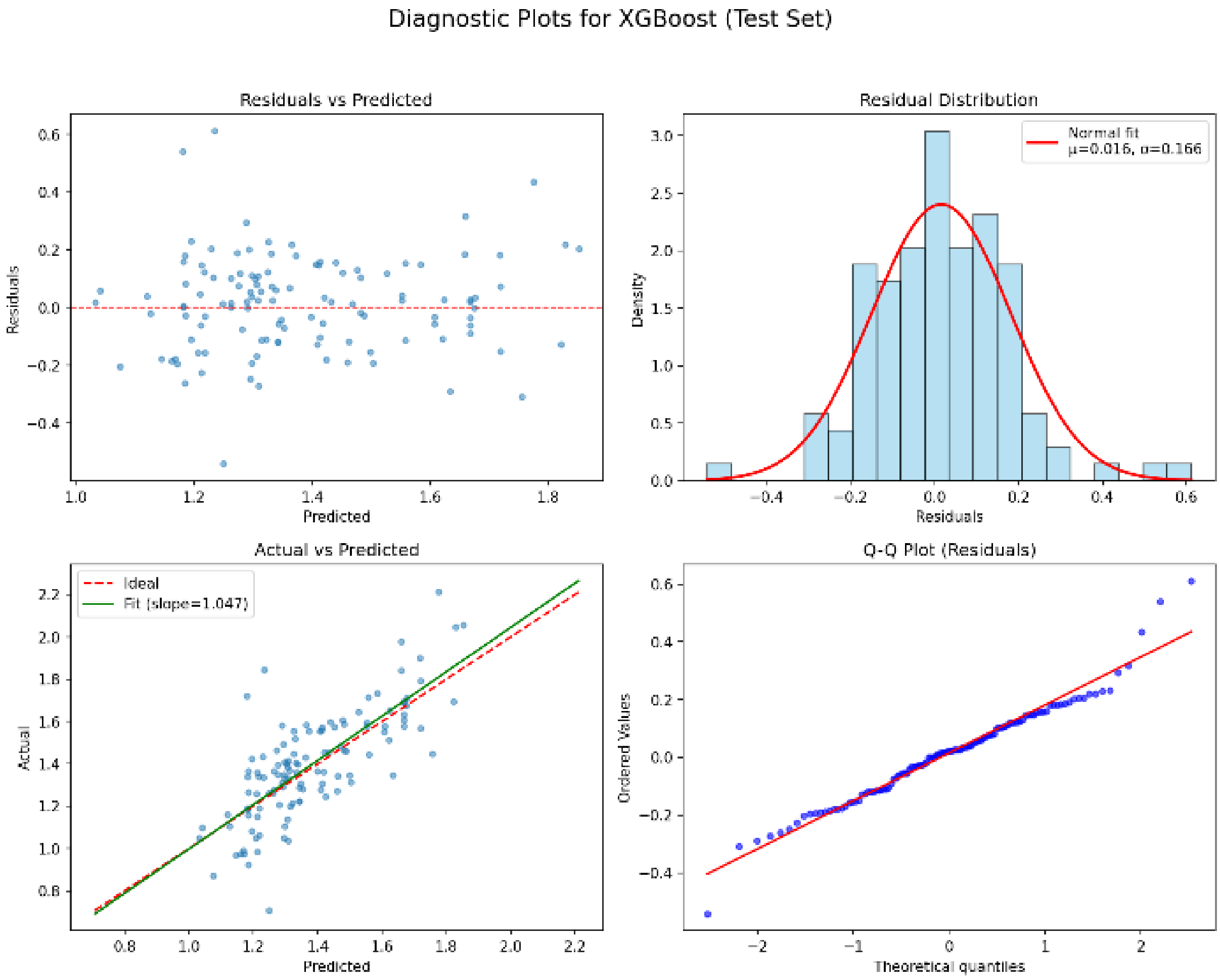

Supplementary Figure S1. Calibration assessment on the XGBoost model.

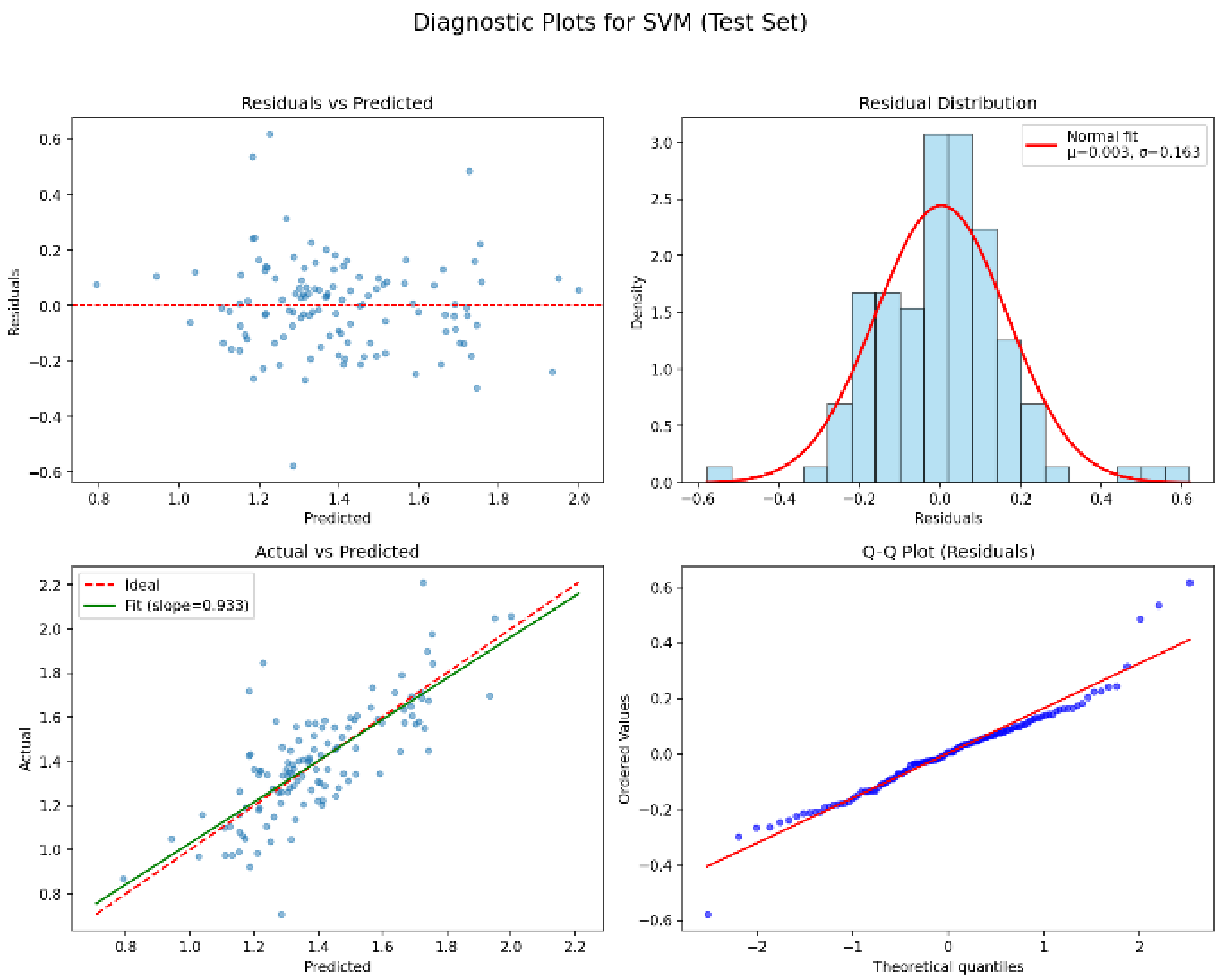

Supplementary Figure S2. Calibration assessment on the SVM model.

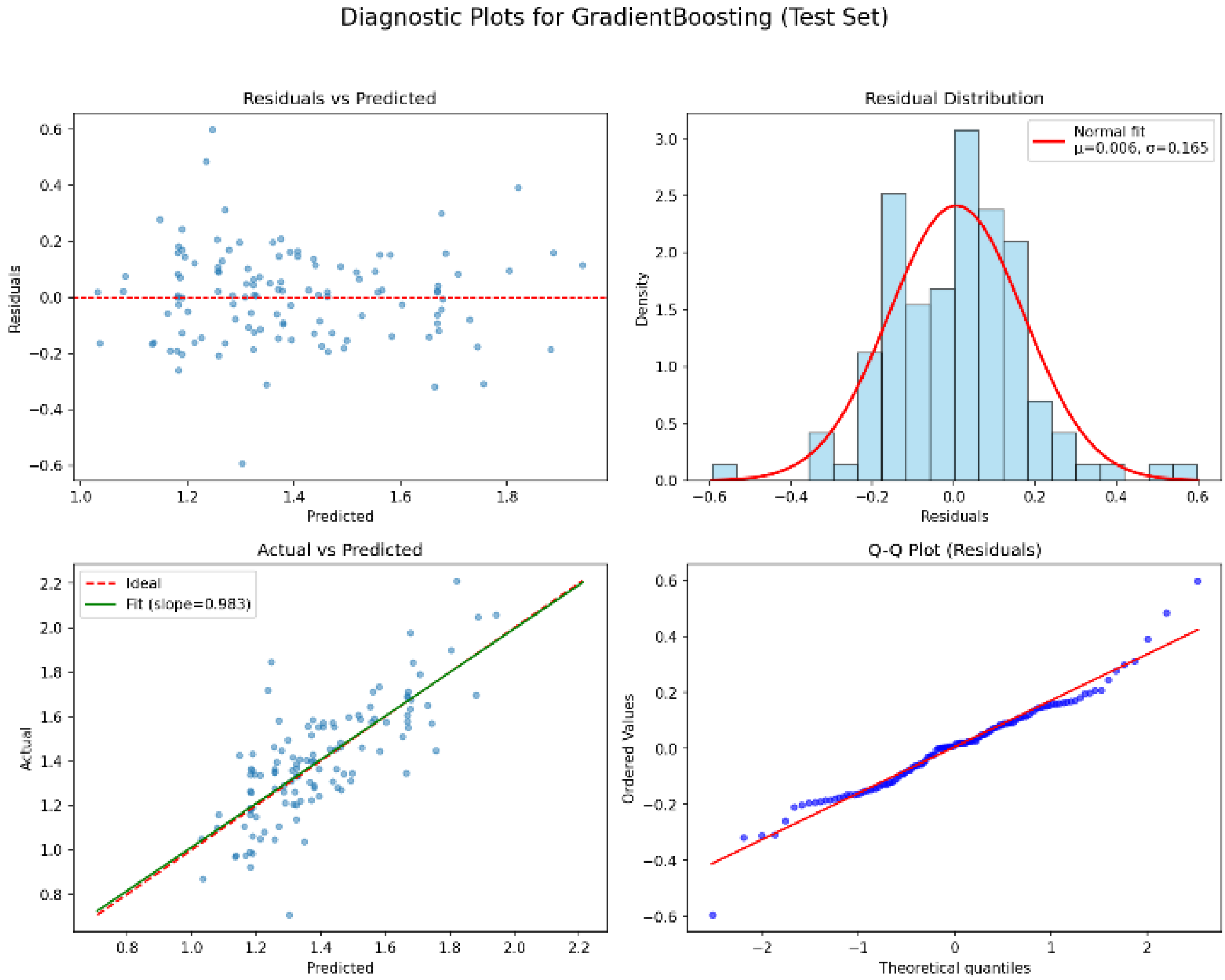

Supplementary Figure S3. Calibration assessment on the GradientBoosting model.
