# Supplementary material for: Development and validation of predictive models for the hemodialysis adequacy index (Kt/V) using multiple linear regression and machine learning algorithms
Source: Front Digit Health. 2026 Oct 12;8:1771806. doi: 10.3389/fdgth.2026.1771806 (PMC13699731; doi:10.3389/fdgth.2026.1771806)
Supplement: Supplementary file 2 [file Table1.docx]

**Supplementary Table S1. The full hyperparameters search spaces.**

param_grids = {

'MLR': {},

'XGBoost': {

'n_estimators': [100, 200],

'max_depth': [3, 5, 7],

'learning_rate': [0.01,0.05, 0.1],

'subsample': [0.8, 1.0],

'colsample_bytree': [0.8, 1.0],

'gamma': [0, 0.1],

'reg_lambda': [0, 0.1, 1.0],

'min_child_weight': [1, 3]

},

'LightGBM': {

'n_estimators': [100, 200],

'num_leaves': [15, 31],

'learning_rate': [0.01, 0.05],

'subsample': [0.8, 1.0],

'colsample_bytree': [0.8, 1.0],

'reg_alpha': [0, 0.01],

'reg_lambda': [0, 0.01]

},

'RandomForest': {

'n_estimators': [100, 200, 300],

'max_depth': [5, 10, 15, 20],

'min_samples_split': [2, 5, 10],

'min_samples_leaf': [1, 2, 4],

'max_features': ['sqrt', 'log2', None]

},

'GradientBoosting': {

'n_estimators': [100, 200, 300],

'learning_rate': [0.01, 0.05, 0.1],

'max_depth': [3, 5, 7],

'subsample': [0.7, 0.85, 1.0],

'min_samples_split': [2, 5, 10],

'min_samples_leaf': [1, 2, 4]

},

'KNN': {

'n_neighbors': [3, 5, 7, 9],

'weights': ['uniform', 'distance'],

'p': [1, 2]

},

'SVM': {

'C': [0.1, 0.5, 1, 5, 10, 50],

'gamma': ['scale', 0.01, 0.05, 0.1, 0.5],

'kernel': ['rbf'],

'epsilon': [0.01, 0.05, 0.1, 0.2]

},

'BPNN': {

'hidden_layer_sizes': [(5,), (5, 3), (8,)],

'activation': ['tanh'],

'alpha': [0.001, 0.01, 0.05],

'learning_rate_init': [0.001, 0.005]

}

}
